# Immunization with Pooled Antigens for *Clostridium perfringens* Conferred Partial Protection against Experimental Necrotic Enteritis in Broiler Chickens

**DOI:** 10.3390/vaccines10060979

**Published:** 2022-06-20

**Authors:** Baohong Yuan, Zhifeng Sun, Mingmin Lu, Hyun Lillehoj, Youngsub Lee, Liheng Liu, Xianghe Yan, Danchen Aaron Yang, Charles Li

**Affiliations:** 1 Animal Bioscience and Biotechnology Laboratory, Agricultural Research Service (ARS), United States Department of Agriculture (USDA), Beltsville, MD 20705, USA; yuanbaohong@gdpu.edu.cn (B.Y.); zhifeng.sun@usda.gov (Z.S.); mingmin.lu@hotmail.com (M.L.); hyun.lillehoj@usda.gov (H.L.); youngsub.lee@usda.gov (Y.L.); liulh0714@jxau.edu.cn (L.L.); 2 School of Basic Medicine Sciences, Guangdong Pharmaceutical University, Guangzhou 510006, China; 3 Environmental Microbial and Food Safety Laboratory, Agricultural Research Service (ARS), United States Department of Agriculture (USDA), Beltsville, MD 20705, USA; xianghe.yan@usda.gov; 4 College of Veterinary Medicine, Nanjing Agricultural University, Nanjing 210095, China; d.a.yang@njau.edu.cn

**Keywords:** *Clostridium perfringens*, vaccine candidates, NetB, phospholipase C, fructose-1,6-bisphosphate aldolase, zinc metalloprotease, collagen adhesion protein

## Abstract

Necrotic enteritis (NE) is a multifactorial and important enteric infectious disease etiologically caused by pathogenic *C. perfringens* infection, accounting for the estimated loss of around USD 6 billion in the global poultry industry. The increasing incidence of NE was found to be associated with the voluntary reduction or withdrawal of antibiotic growth promoters from animal feed during recent years. Therefore, the development of effective vaccines specific to NE assumes a priority for the poultry industry. This study aimed to identify the potential *C. perfringens* proteins as vaccine targets for NE. Three recombinant *C. perfringens* proteins targeting five antigens were prepared: two chimeric proteins (alpha-toxin and NetB, fructose-1,6-bisphosphate aldolase (FBA) and a zinc metalloprotease (Zm)), and one single collagen adhesion protein (Cna). Their protection efficacies were evaluated with a potent challenge model of *Eimeria maxima*/*C. perfringens* dual infections using a *netB^+^tpeL^+^* *C. perfringens* strain. Young chicks were immunized twice subcutaneously with adjuvanted *C. perfringens* proteins on Days 4 and 15. At six days after the second immunization, the chickens immunized with Cna, FBA, and Zm antigens, and alpha-toxin had much higher serum antibody titers than unvaccinated controls prior to the challenge. Following the challenge, the pooled antigen-immunized group demonstrated no mortality and the least lesion scores against virulent challenge. The results indicate that the immunization with multicomponent antigens, including *C. perfringens* housekeeping protein Cna, may confer partial protection.

## 1. Introduction

*Necrotic enteritis* (NE) is an important enteric disease, responsible for the annual loss of around 6 billion US dollars to global poultry producers [1]. NE is a complex and multifactorial disease etiologically caused by the pathogenic *C. perfringens,* the isolates of which are classified into six types (A-G) based on the major toxins produced [2,3,4]. Other key risk factors favoring the growth of *C. perfringens* may also enhance the development of NE, such as the pre- or co-infection with coccidiosis, nutritional factors (diets enriching animal proteins, high energy, wheat- or barley-based components), husbandry mismanagement, or bird immunosuppression [5,6]. NE has been well-controlled by the application of antibiotic growth promoters (AGP) and ionophore coccidiostats in the feed in the past few decades [5]. However, a resurgence in NE incidence has been found to be associated with voluntary reduction or complete withdrawal of these AGPs from feed [7]. There are increasing pressures from public health concerns and regulatory agencies over the emergence of multidrug-resistant bacteria or the presence of AGP residual pollution in the food products or environment. Ideally, vaccination as an alternative approach to antibiotics would assume high priority. Only one live *Salmonella*-vector vaccine (RASV) expressing *C. perfringens* genes coding for an a-toxin fragment and NetB toxin (AVERT^R^ NE) has recently been commercially available (http://www.huvepharma.us/product/avert-ne/) (accessed on 27 May 2022)).

To control the NE, scientists have made great efforts toward understanding the pathogenesis of *C. perfringens* and developing vaccines against necrotic enteritis in broiler chickens. Multiple vaccine platforms have been tested, including toxoids, whole inactivated vaccines, live-attenuated vaccines, subunit vaccines, recombinant vectored vaccines, or attenuated *Salmonella*-vectored vaccines [8,9,10,11]. Among them, some vaccine targets have been reported for recombinant subunit vaccines. Alpha-toxin (CPA or phospholipase C fragment of CPA, Plc) is an essential protein proposed for disease processes [12], but the role of CPA may be limited since the engineered *cpa* mutants are found to still maintain full virulence in vivo [13]. Later, the presence of necrotic enteritis B-like toxin (*netB)* gene in Type G (*plc^+^netB^+^*) strains has broadly been shown to be essential for NE pathogenicity [2,14]. In addition to NetB toxin, other virulence factors or virulence-associated factors have also been studied. Fructose-1,6-bisphosphate aldolase (FBA) is an enzyme involved in the Embden–Meyerhof–Parnas glycolytic pathway and in gluconeogenesis [15]. Zinc metalloproteases (Zm) are zinc-binding proteases shown to degrade mucin, the primary constituent glycoprotein of the mucosa, and have been implicated to be involved in the pathogenesis of necrotic enteritis [16]. Collagen-binding protein A (Cna) and fibrinogen-binding proteins FbpA and FbpB are implied to function as adhesins during infection. They are highly associated with the production of a sortase-dependent pilus by *C. perfringens* and play critical roles in collagen binding and NE pathogenesis [17]. Among these recombinant subunit vaccine targets, various levels of protection against NE have been reported in vaccine studies using the single subunit versions of CPA toxin, NetB toxin, FBA, pyruvate ferredoxin oxidoreductase (PFOR), and elongation factor Tu [18,19,20,21]. Understanding the pathogenesis of *C. perfringens* is vital for full NE prevention and control. It appears that several factors, such as NetB, Zm, and Cna, are more likely expressed in virulent *C. perfringens* strains causing necrotic enteritis than avirulent strains based on preliminary findings [16,17,21,22]. Interestingly, NetB, Plc, and FBA are suggested to be probably expressed on the *C. perfringens* surfaces, and FBA may serve as an adhesin function [10,15]. Immunization with mucosally vectored *Salmonella* expressing these triple antigens (NetB, Plc, and FBA) conferred partial protection [10]. Zinc metalloprotease was initially reported to be immunogenic as a hypothetic protein [21], and was found to contribute to the virulence of *C. perfringens* strains that cause avian NE [16]. Mutant generation via disruption of the *zm* gene using mutagenesis was shown to significantly reduce the virulence of *C. perfringens*, suggesting its role in disease pathogenesis [16]. Collagen adhesion protein as a pilus component of *C. perfringens* has been shown to be associated with the adherence of *C. perfringens* to the host in pathogenesis study, and inactivation of the pilus genes resulted in inhibition of pilus production, highly reducing the capability of *C perfringens* to bind collagen and initiate disease [17,23,24]. We hypothesized that the pooled recombinant immunogens from these key targets could offer better protection against NE challenges. The objective of this study was to prepare recombinant proteins targeting five virulence factors or virulence-associated factors of *C. perfringens* in either single (Cna) or chimeric forms (NetB-CPA and FBA-Zm), and compare the vaccine efficacies via subcutaneous immunization in a well-established NE challenge model, a dual infection model with *Eimeria maxima* pre-infection, followed by pathogenic *C. perfringens* co-infection.

## 2. Materials and Methods

### 2.1. Design and Construction of Recombinant Chimeric NetB-CPA (NA) and FBA-Zm (FZ), and Cna Expression Vector

The specific fragment gene sequences from NetB toxin and Plc portion of alpha-toxin (CPA) were fused as chimeric recombinant NA using alpha-helix forming linkers A(EAAAK)_4_A, as similarly described elsewhere [25], while the *fba* gene and zinc metallopeptidase *zm* gene were used to construct chimeric *fz* gene using the same alpha-helix forming linker, as previously described [26]. The *cnaA* gene was used as a single construct using the same sequence as described elsewhere [27]. All these genes were codon-optimized and synthesized by Synbio Inc. (Synbio Technologies LLC, Monmouth Junction, NJ, USA). EcoRI and HindIII restriction sites were inserted at 5′ and 3′ ends of each construct, which were later subcloned into the EcoRI and HindIII restriction sites of the pET-20b(+) vector (Novagen, Madison, WI, USA).

### 2.2. Expression and Purification of Recombinant C. perfringens Proteins

The synthesized plasmids were transformed into BL21-AI *E. coli* competent cells (Thermo Fisher Scientific, Waltham, MA, USA) on LB agar plates (Research Products International Inc., Mt Prospect, IL, USA) supplied with 100µg/mL of ampicillin (Sigma-Aldrich, St. Louis, MO, USA). The positive bacterial strains containing the recombinant plasmids were characterized. Bacterial culture was incubated at 37 °C with shaking at 225 rpm overnight under the induction of 0.25 mM isopropyl-β-D-thiogalactopyranoside (IPTG, Sigma-Aldrich, St. Louis, MO, USA) in LB media containing 100 µg/mL of ampicillin (Sigma-Aldrich, St. Louis, MO, USA). The expressed recombinant proteins were purified with the Ni-NTA Agarose based on the manufacturer’s product instruction (QIAGEN Inc., South San Francisco, CA, USA). Quality control was carried out with an anti-His tag antibody (Thermo Fisher Scientific, Waltham, MA, USA) using Western blot analysis. Briefly, bacteria were harvested by centrifugation at 15,000× *g* for 10 min at 4 °C. The supernatants were removed, and cell pellets were resuspended with 1× PBS. Bacterial samples were sonicated for 10 min and centrifugated at 15,000× *g* for 20 min at 4 °C, and the pellets were collected and resuspended in 8M Urea at room temperature for over 5 h or overnight. Inclusion body samples containing the recombinant proteins were centrifugated at 15,000× *g* for 30 min at 15 °C. Ni-NTA resin/1× PBS was added to the supernatants, and tubes were incubated on a shaker for 1 hour (hr). Samples were then centrifugated at 1100× *g* for 5 min, and resins were collected and washed with 1× PBS, 15 mL of 0.1 M Tris-HCl (pH 7.4), and 15 mL of 0.1 M Tris-HCl (pH 8.0), respectively. The recombinant protein was eluted from the column with 0.25 M Imidazole in PBS and dialyzed against three changes in PBS with stirring at 4 °C. The purified protein was passed through the Detoxi-Gel Endotoxin Removing Columns (ThermoFisher Scientific, Waltham, MA, USA) to remove endotoxin, and protein concentration was measured using Bradford Reagent (Sigma-Aldrich, St. Louis, MO, USA). The molecular weight and purity of the purified proteins were determined with SDS–PAGE and Western blot analysis.

### 2.3. Bacterial Strain

The LLN_Tpel17 *C. perfringens* isolate (simplified as Tpel17) were prepared as described elsewhere [28,29]. Briefly, the Tpel17 isolate was first cultured in chopped meat glucose medium for 24 hr in anaerobic chambers that utilized a gas packet (Mitsubishi Gas Chemical Company, New York, NY, USA) to generate anaerobic conditions (O_2_ < 2%, CO_2_ = 9–13%). The bacteria culture was then inoculated in 150 mL of BYC medium (1:100 dilution) containing Bacto^TM^ Brain–Heart Infusion broth (BHI; Becton Dickinson and Company, Sparks, MD, USA), yeast extract, and Cysteine (Sigma-Aldrich, St. Louis, MO, USA), and cultured for 18 hr at the same anaerobic conditions.

### 2.4. Broiler Chick Husbandry and Experimental Design

One-day-old Ross 708 broiler chicks obtained from Longenecker’s Hatchery (Elizabethtown, PA) were housed in Petersime starter brooder units and provided with feed and water *ad libitum*. A total of 91 one-day-old chicks were randomly assigned to 7 groups, with 13 birds per group: naïve sham control (N), *E. maximum*/*C. perfringens* (EMCP) challenge control, adjuvant control (Adj), Cna-immunized group (Cna), FZ-immunized group (FZ), NA-immunized group (NA), and pooled antigen group (Pooled) (Table 1). Chickens were transferred into large hanging cages at the age of 2 weeks and were fed a nonmedicated starter diet containing 16% crude protein and 61% carbohydrate prior to *C. perfringens* infection, and then a standard grower diet containing 24% crude protein and 54% carbohydrate after *C. perfringens* infection, as described elsewhere [19,30,31]. All diets contained 2.4% fiber, 4.7% fat, and 15% vitamin and mineral mixture and were prepared at USDA-Feed Mill (Beltsville, MD). All experiments were approved by the local Institutional Animal Care and Use Committee and performed in animal facilities at Beltsville Agricultural Research Center (Animal Use Protocol No 17-027).

### 2.5. In Vivo Evaluation of Vaccine Efficacy of Recombinant C. perfringens Proteins

Chickens were vaccinated subcutaneously with recombinant proteins adjuvanted with Montanide ISA VG 71 (Seppic Inc., Puteaux, France) on Day 4 (100 µg proteins for the first immunization) and D15 (boost with 50 µg proteins) according to the manufacturer’s product instruction [19,32,33] (Table 1). The pooled antigen groups were immunized with a total of 75 µg of pooled antigens (mixture of NA/FZ/Cna 25 µg each for the first dosing) and boosted with 37.5 µg (12.5 µg of each antigen for the second dosing). Six days after the last immunization, birds were bled from the wings for antibody determination via ELISA. Seven days after the second immunization, chickens were challenged with 5 × 10^3^ sporulated oocysts of *E. maxima* (EM) per bird by oral gavage, then followed by inoculation of *C. perfringens* Tpel17 of around 1 × 10^9^ colony forming units per bird (CFU, all bacteria from the same overnight broth culture in a large flask for the entire trial) by oral gavage at 4 days after *E. maxima* inoculation (Table 1). Two days after the *C. perfringens* infection, birds were euthanized by cervical dislocation (Day 28). The jejunum lesion scores were determined with a section of around 10 cm length flanking Meckel’s diverticulum region, as described elsewhere [19,30]. The lesion scores were evaluated on a scale from 0 (no lesions) to 4 (severe lesions), and those in dead chickens were considered as 4 points in the weighted lesion scoring [34].

### 2.6. Determination of Antibody Titers in Vaccinated Birds by ELISA

Purified Cna and Plc, or chimeric FZ proteins (1 μg/mL in 1 × PBS), respectively, as coating antigens, were added to 96-well ELISA plates and incubated at 4 °C overnight. Plates were washed once and blocked with 200 μL of blocking buffer (1 × PBS/0.1% Tween-20 detergent (Sigma-Aldrich, St. Louis, MO, USA)) at 37 °C for 1 hr. After blocking, 100 μL of serially diluted chicken serum was added to each well of the plate for incubation at 37 °C for 1 hr. Plates were then washed four times, and 100 μL of diluted HRP-conjugated goat anti-chicken IgY antibody (Sigma-Aldrich, St. Louis, MO, USA) was added to each well for incubation at 37 °C for 30 min. Following four washes, 100 μL of TMB Reagent (GenScript, Piscataway, NJ, USA) was added to each well and incubated at room temperature for 15–20 min. Lastly, 100 μL of stop solution (2N H_2_SO_4_) was added to the wells and the OD_450_ value was read using a microplate reader. Antibody titers were determined as the reciprocals of dilution folds of serum samples, in which OD450 reached around 0.5 with ELISA.

### 2.7. Statistical Analysis

The mortality rate in each group was estimated using sample proportion with the 95% confidence interval (95% CI), computed using a numerical method based on the likelihood function of the number of deaths (modeled as a realization of a binomial random variable). The difference in mortality rates among the seven groups was tested using the Fisher’s exact test (developed by Ronald A. Fisher, London, England, UK) in R (version 4.1.2). Since the number of groups was larger than two, the *p*-value computed via Monte Carlo simulation was compared with the *p*-value calculated using the analytical approach. The antibody titers were analyzed using a one-way ANOVA in SAS 9.4 for Windows followed by Duncan’s multiple range tests (Cary, NC, USA). All of the data are expressed as mean ± SEM for each treatment. The lesion scores were analyzed using a Kruskal–Wallis Test among the groups and the Mann–Whitney U test between two groups [10,11]. Differences were considered statistically significant at *p* ≤ 0.05.

## 3. Results

### 3.1. Mortality Post-Challenge in Vaccinated Groups

**Figure 1:**
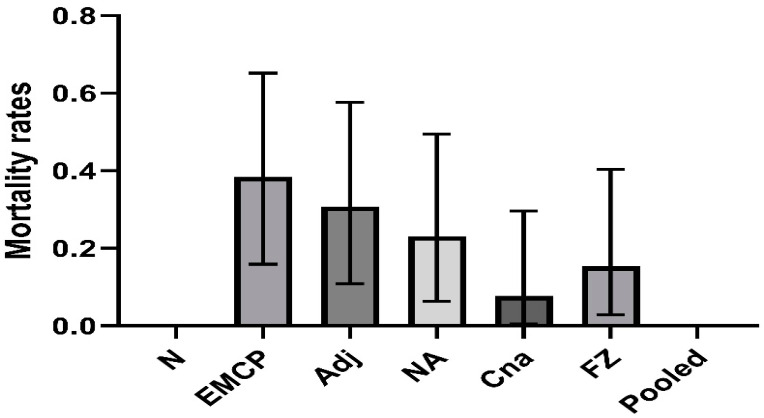
Mortality rates with 95% confidence intervals of vaccinated broiler chickens following necrotic enteritis challenges. Birds were immunized with recombinant proteins on Day 4 and Day 15: EMCP = positive challenge control; Adj = adjuvant + EMCP challenge; NA= chimeric NetB and Plc; FZ = chimeric FBA and Zm; Cna = collagen-binding protein; Pooled = pooled NA, FZ, and Cna antigens. The birds with 13 birds per group were then challenged with *Eimeria maxima* (EM, 5 × 10^3^ oocysts/bird, oral gavage) on Day 22, followed by CP *netB^+^tpel^+^* LLY_Tpel 17 (1 × 10^9^ cfu /bird, oral gavage) on Day 26. Mortalities were recorded after *C. perfringens* challenge.

Figure 1 shows the mortality rates in control and vaccinated groups. Fisher’s exact test suggested that the mortality rates were significantly different among the groups (*p* = 0.036 with Monte Carlo simulation). In the *E. maxima*/*C. perfringens* challenge control group, 0.385 mortality (5 dead out of 13) was observed. Meanwhile, 0.308 mortality was found in the adjuvant control group (4 dead out of 13). There were lower mortalities in vaccinated groups: 0.231 mortality for the NA-immunized group (3 dead out of 13), 0.154 mortality for the FZ-immunized group (2 dead out of 13), and 0.077 mortality for the Cna-immunized group (1 dead out of 13) (Figure 1). No mortality was seen in the pooled antigens-immunized group, indicating that these antigens were likely to provide good protection against the lethal challenge of *E. maxima* and *C. perfringens*.

### 3.2. Jejunum Lesion Scores

**Figure 2:**
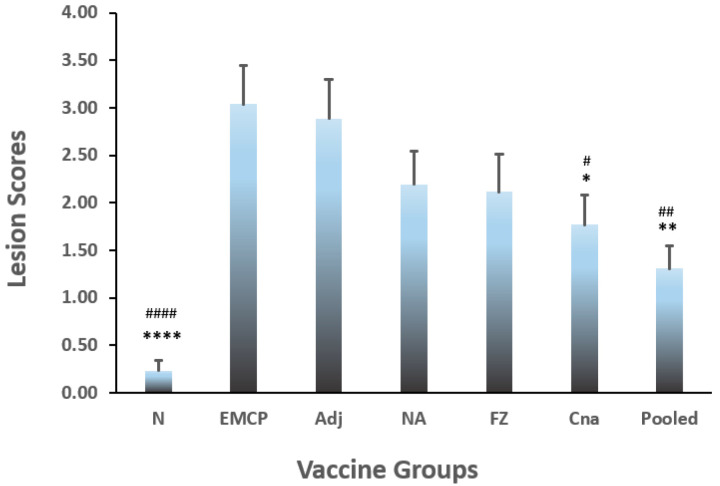
Lesion scores in vaccinated broiler chickens following necrotic enteritis challenge. The statistical differences were analyzed by the Kruskal–Wallis test and the Mann–Whitney U test. The chicks with 13 birds per group were immunized with recombinant proteins on Day 4 and Day 15: EMCP = positive challenge control; Adj = adjuvant + EMCP challenge; NA = chimeric NetB and Plc; FZ= chimeric FBA and Zm; Cna= collagen-binding protein; Pooled = pooled NA, FZ, and Cna antigens. The vaccinated birds were then administrated with *Eimeria maxima* (EM, 5 × 10^3^ oocysts/bird, oral gavage) on Day 22, followed by *C. perfringens* (CP) *netB^+^tpel^+^*LLY_Tpel 17 strain (1 × 10^9^ cfu /bird, oral gavage) on Day 26. Birds were sacrificed on Day 2 post-CP infection, and jejunum lesion scores were determined. The * or # marks show statistically significant difference (* *p* ≤ 0.05; ** *p* ≤ 0.01; **** *p* ≤ 0.0001) when compared with the value in Adj group, while ^#^ *p* ≤ 0.05; ^##^ *p* ≤ 0.01; ^####^ *p* ≤ 0.0001 when compared with the value in EMCP group, respectively).

Intestine lesion scores were also determined on Day 2 post-*C. perfringens* infection. The Kruskal–Wallis test indicated there was a significant difference among the groups (*p* < 0.0001). Post hoc analysis was further conducted with the Mann–Whitney U test between independent groups. Figure 2 showed the lesion scores in all the experimental groups. Compared with the control groups, all of the vaccination groups had lower lesion scores. However, a significant statistical difference in lesion scores was observed between pooled antigen group and controls groups (both EMCP and adjuvant control) (*p* < 0.01), and between the Cna group and either control group (adjuvant or EMCP) and (*p* < 0.05), while no significant difference was detected between either control (EMCP or Adjuvant) and other vaccinated groups (*p* > 0.05). No significant difference was found between EMCP and adjuvant control groups.

### 3.3. Determination of Antibody Titers

**Figure 3:**
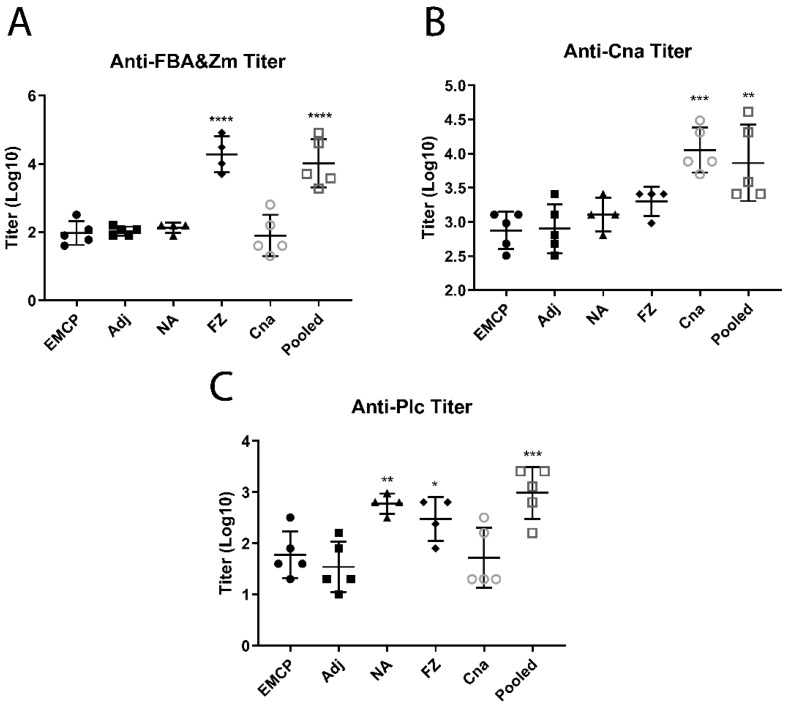
The antibody titers in control and vaccination groups determined via ELISA: (**A**) anti-FZ (FBA-Zm) titers; (**B**) anti-Cna titers; (**C**) anti-Plc titers. Antibody titers were determined using log_10_ (reciprocals of dilution folds of serum samples), which OD450 reached around 0.5 with ELISA. The recombinant FZ (chimeric FBA-Zm), Cna, or Plc proteins were used as coating antigens. EMCP = positive challenge control; Adj = adjuvant + EMCP challenge; NA = chimeric NetB and Plc; FZ= chimeric FBA and Zm; Cna = collagen-binding protein; Pooled = pooled NA, FBA, and Cna antigens. The * marks show statistically significant differences (* *p* ≤ 0.05; ** *p* ≤ 0.01; *** *p* ≤ 0.001; **** *p* ≤ 0.0001) when compared with the value in adjuvant (Adj) control group.

After immunization with recombinant proteins, specific antibodies were produced in these chickens. On Day 6 after the second immunization (Day 21), chickens were bled to determine antibodies prior to the NE challenge. As indicated by Figure 3, large amounts of anti-FZ (chimeric FBA-Zm) antibodies were generated in the single FBA-Zm antigen and pooled antigen groups, which were both immunized with chimeric FBA-Zm (Figure 3A), where 2 log_10_ difference in titers existed between the adjuvant control group and group with FBA-Zm immunization (*p* ≤ 0.0001 for either single or pooled antigens), while higher antibodies were also produced in single Cna and the pooled antigen groups, which were immunized with Cna antigens, where about less than 1 log_10_ difference existed between the adjuvant group and Cna group (*p* ≤ 0.001) or between the adjuvant group and pooled antigen group (*p* ≤ 0.01) (Figure 3B). There were also higher anti-CPA (Plc) antibodies in the single NA immunized group or pooled antigen group with a significant difference (*p* ≤ 0.01 for NA vs. Adj group; *p* ≤ 0.001 for pooled antigens vs. Adj group) (Figure 3C). Interestingly, the FZ-immunized group was found to have some binding activities with the Plc antigens coated on the ELISA plate, which was most possibly caused by the nonspecific binding with common His-tag (other than with FBA or Zm epitopes). All the antigens were His-tagged, so that a high level of anti-FZ antibodies may interfere with the anti-Plc readings when the plate was coated with His-tagged Plc.

## 4. Discussion

Necrotic enteritis is a serious enteric infectious disease for chickens that may cause sudden death with up to 50% mortality (acute form) or may result in mucosal damage to intestines, leading to necrosis, ulceration, inflammation, reduced weight loss, and carcass condemnation (the most common subclinical form) [5,6]. Therefore, NE is an economically important disease that has a significant adverse impact on bird welfare and profitability for the poultry producer with an estimated economic loss of around USD 6 billion annually to the global poultry industry [1]. Vaccination is a good antibiotic alternative strategy as a preventive measure. The availability of an effective NE vaccine could improve poultry well-being and profitability for the poultry producers. 

NetB, Plc, Cna, FBA, and Zm are important virulence factors or virulence-associated factors, which play critical roles in bacterial adhesion to the host, localization, energy utilization, and spread in the early infection. Vaccination with these recombinant proteins may help ameliorate the severity of the NE challenge in chickens. The potent challenge model was utilized with *E. maxima* pre-infection, followed by pathogenic *C. perfringens* Tpel17 inoculation. This study was carried out to evaluate the protection efficacies of three types of recombinant *C. perfringens* proteins adjuvanted with ISA71VG using a well-established experimental NE model. Understanding the roles of these virulence factors and their associated factors in pathogenesis may help design optimal vaccines to efficiently disrupt the phases of transmission, colonization, and proliferation of *C. perfringens* during the early infection stage.

In this study, several encouraging results were found: (1) Chickens immunized with all adjuvanted proteins post potent challenge had reduced mortalities with no mortality in the pooled antigen group; (2) chickens immunized with the adjuvanted pooled proteins post potent challenge had significantly reduced lesion scores, compared with the challenge control or infected and adjuvant groups; (3) much higher anti-FZ antibody titers were observed in the FZ-immunized and pooled antigens groups, and higher anti-Cna antibody titer in Cna-immunized and pooled antigen groups, when compared with adjuvant control or challenge control group; (4) mortality rates were very close between the adjuvant control and challenge control groups after potent challenge, implying that immunization with only ISA71VG adjuvant may not appear to have a protective effect against NE, in agreement with previous observation [19].

In this study, it seems that robust immune responses were observed by the immunizations with chimeric FZ protein or with Cna at a magnitude level. The molecular sizes are as follows: in chimeric FBA-Zm and Cna, the size was around 70 kD, while in chimeric NA, it was around 50 kD. More epitopes on the large FBA-Zm or Cna protein molecules may induce the host to generate more antibodies. On the other hand, FBA and Zm were identified via mass spectrophotometry to be two out of six secreted proteins unique to virulent strains that were highly immunoreactive to serum antibodies from immune birds by Prescott’s group [21]. As the gut mucosa integrity is disrupted by the predisposing factors (such as *Eimeria spp.* infection), *C. perfringens* outgrowth and penetration of the intestinal mucosa may lead to either mild mucosal damage or even extensive necrosis by accompanying secretion of a series of mucolytic enzymes, pore-forming toxins such as NetB, and tissue-degrading toxins (such as α-toxin and TpeL) [35]. Induction of robust neutralization antibodies against these toxins, other virulence factors and virulence-associated factors resulting from the systemic vaccination *via* a subcutaneous route may help establish a regulatory environment to reduce bacteria spreading and ameliorate these factors’ negative effects. The ideal vaccines should elicit both mucosal and systemic immune responses against mucosal pathogens such as *C. perfringens* [5,10,11,35].

In this study, the immunization with Cna protein resulted in less mortality (7.7%) post-NE challenge compared with higher mortality in the positive challenge control group (38.5%) and adjuvant/infection control (30.8%) and had less intestinal lesion score than the adjuvant control group. The additive contribution effect of the Cna factor was evident in the mortality rate. Gong’s group demonstrated that subcutaneous triple immunization with CnaA or another pilus subunit FimB conferred partial protection against the onset of NE by significantly reducing the NE lesions when compared with the adjuvant control [27]. Of course, multiple vaccinations by laborious intramuscular injection are not practical in the field of the poultry industry. The ideal immunogen delivery route in mass vaccination should be mucosal one or *in ovo* one in broiler chickens. The pooled antigen group had no mortality in this study, which is supportive of the notion that vaccination with a single immunogenic component does not provide full protection against a complex NE, fully echoing other reports [20,36,37]. Immunizations with multiple immunogens and other practical mass vaccination means should be considered in the design of the NE vaccine. As discussed earlier, FBA, Zm, Cna, NetB, and Plc are good vaccine candidates against NE in chickens. In fact, several chimeric proteins containing multiple immunogenic components have been reported in design, such as chimeric proteins NetB-Plc-Tpel [38], NetB-Plc-Zm [39], and Plc-FBA-NetB [10], and some may confer partial protection in chickens. Future studies will focus on a better vector or route to deliver these multiple immunogenic targets. It should be mentioned that the combination of these antigens may need repetition before future studies are performed.

In summary, three *C. perfringens* recombinant proteins were founded to elicit varying levels of protective immunity in an experimental NE challenge model. Multiple immunogenic components should be considered for the development of an effective vaccine to control NE. Future studies merit further investigation into these critical proteins to define their molecular mechanisms for the control of NE.

## Figures and Tables

**Table 1:** Experimental design of vaccination studies *.

Group No.	Group Name	Bird Number	Vaccination (D4, D15)	Challenge		Sacrifice (D28)
				EM(D22)	CP (D26)	
**1**	Naive	13	PBS	PBS	BYC	S
**2**	Challenge Control (EMCP)	13	PBS	EM	CP	S
**3**	Adjuvant control (Adj)	13	Adj + PBS	EM	CP	S
**4**	Cna	13	Adj + Cna	EM	CP	S
**5**	FZ	13	Adj + FZ	EM	CP	S
**6**	NA	13	Adj + NA	EM	CP	S
**7**	Pooled (NA + FZ + Cna)	13	Adj + NA + FZ + Cna	EM	CP	S

* Vaccinated chickens were challenged with *E. maxima* (EM, 5 × 10^3^ oocysts/bird orally on Day 22), followed by oral gavage of *C. perfringens* (CP, 1 × 10^9^ CFU at D26). BYC = BYC medium; PBS = phosphate-buffered saline; S = sacrificed; EMCP = positive challenge control; Adj = adjuvant + EMCP challenge; NA= chimeric NetB and Plc; FZ = chimeric FBA and Zm; Cna = collagen-binding protein; Pooled = pooled NA, FZ, and Cna antigens.

## Data Availability

The data presented in this study are available within the article.

## Abbreviations

NE	Necrotic enteritis
CPA	Alpha-toxin
Plc	Phospholipase C (PLC) enzyme of alpha-toxin
NetB	Necrotic enteritis B-like toxin
FBA	Fructose-1,6-bisphosphate aldolase (FBA)
Zm	Zinc metalloprotease (Zm)
Cna	Collagen adhesion protein
AGP	Antibiotic growth promoters
EM	*Eimeria maxima*
CFU	Colony-forming units
